# Survival Impact and Risk Factors of Cervical Lymph Node Metastasis in Maxillary Gingival Squamous Cell Carcinoma: A Retrospective Study of 81 Patients

**DOI:** 10.1002/hed.70021

**Published:** 2025-08-30

**Authors:** Takeshi Kuroshima, Naoya Kinoshita, Naoto Nishii, Yu Oikawa, Takuma Kugimoto, Hideaki Hirai, Hirofumi Tomioka, Yasuyuki Michi, Hiroyuki Harada

**Affiliations:** ^1^ Department of Oral and Maxillofacial Surgical Oncology Graduate School of Medical and Dental Sciences, Institute of Science Tokyo Tokyo Japan; ^2^ Division of Oral and Maxillofacial Surgery, Faculty of Dentistry & Graduate School of Medical and Dental Sciences Niigata University Niigata Japan

**Keywords:** cervical lymph node metastasis, maxillary gingiva, oral cancer, prognosis, squamous cell carcinoma

## Abstract

**Background:**

This study aimed to investigate the survival impact and predictive factors of cervical lymph node metastasis (CLNM) in patients with maxillary gingival squamous cell carcinoma (MGSCC).

**Methods:**

This retrospective study included 81 patients with MGSCC who underwent radical surgery from 2008 to 2018.

**Results:**

Pathological CLNM was observed in 24 patients, prevalently in levels IB and II on the ipsilateral side. Cox proportional hazards model analysis revealed histologic grade, CLNM, and local recurrence as independent prognostic factors for 5‐year disease‐specific survival. Multivariate analysis identified tumoral spread to the gingivobuccal sulcus and the maxillary tuberosity as independent risk factors for CLNM.

**Conclusions:**

CLNM is associated with poor survival in patients with MGSCC. Primary tumors extending into the gingivobuccal sulcus or maxillary tuberosity may predict CLNM, particularly at levels IB and II. These findings may provide valuable insights for optimizing neck management strategies.

## 1 Introduction

Oral cancer is one of the most prevalent malignancies globally, with an estimated 389 485 new cases and 188 230 cancer‐related deaths in 2022 [1]. Squamous cell carcinoma (SCC) is the predominant histopathological type of oral cancer. The tongue and floor of the mouth account for over 50% of oral cancer subsites, whereas SCC arising in the maxillary gingiva is much less common [2]. Consequently, studies involving a large number of maxillary gingival SCC (MGSCC) cases are limited, and comprehensive characterization of its clinicopathological features and identification of prognostic factors remain essential.

Cervical lymph node metastasis (CLNM) is an accurate prognostic factor for patients with oral SCC. Recent studies have indicated that MGSCC exhibits a higher potential for CLNM than previously reported [3, 4, 5, 6, 7, 8]. Several systematic reviews and meta‐analyses have recommended elective neck dissection (END) for patients with clinically node‐negative (cN0) MGSCC; however, its survival benefit remains unclear [9, 10]. Although the wait‐and‐see strategy is a reasonable and widely practiced approach for neck management, there is a possibility that patient survival could be compromised if early detection and treatment of CLNM are not feasible. Therefore, further studies identifying reliable risk factors for CLNM are required to develop appropriate neck management strategies based on such factors in MGSCC patients.

Previous studies that statistically analyzed risk factors for CLNM in MGSCC patients have been limited by small sample sizes due to the rarity of the disease [11, 12, 13, 14, 15, 16, 17, 18, 19]. Moreover, many of these studies included both MGSCC and palatal carcinomas in their analyses [11, 12, 13, 14, 15, 16, 17]. Although the lymphatic collecting vessels from the medial side of the maxillary gingiva overlap with those of the palate, the drainage from the buccal side follows a different pathway [20]. Therefore, to enable a more precise investigation of CLNM, analyzing MGSCC and palatal cancers separately would be appropriate.

The aim of this study was to elucidate the frequency and survival impact of CLNM, and to identify its risk factors in MGSCC patients. To ensure the accuracy of the results, no patients with palatal cancer were included. To the best of our knowledge, among statistical analyses on CLNM risk factors, the present study includes the largest number of MGSCC cases. Consequently, our findings may provide clinicians with more reliable evidence to guide appropriate neck management strategies.

## 2 Materials and Methods

### 2.1 Patients

This study retrospectively reviewed the medical records of 81 patients with MGSCC who underwent radical surgery at the Department of Oral and Maxillofacial Surgery at Tokyo Medical and Dental University Hospital from 2008 to 2018. Cases with metachronous oral malignancies in other subsites were excluded from this study, as the presence of such multiple primary tumors could have influenced metastasis or survival outcomes, potentially confounding the study results. Demographic and clinical data, including age, sex, T stage, N stage, histologic grade, CLNM, invasive features of the primary tumor (bone invasion, maxillary sinus invasion, perineural invasion, and lymphovascular invasion), surgical margin status, local recurrence, preoperative and postoperative treatment, and prognosis were obtained from the patients' medical records. Tumors were clinically staged based on the seventh edition of the American Joint Committee on Cancer TNM staging system [21]. This study complied with the Declaration of Helsinki and obtained approval from the Institutional Review Board of Tokyo Medical and Dental University (D2015‐600). Informed consent was obtained in the form of opt‐out on the in‐hospital bulletin.

### 2.2 Treatments

All patients underwent pretreatment imaging, including computerized tomography (CT), magnetic resonance imaging, ultrasonography (US), and ^18^F‐fluorodeoxyglucose (FDG) positron emission tomography (PET). Primary tumor and CLNMs were assessed based on physical examination and imaging findings. Patients with an advanced tumor (cStage III or IV) who had a protracted waiting period for surgery received preoperative radiation therapy (RT) to the primary tumor. The radiation field included the neck when clinically positive nodes were identified. Preoperative RT was administered with a fraction dose of 2 Gy to a total dose of either 40 or 50 Gy. When tolerated, low‐dose cisplatin (4 mg/m^2^) was concurrently administered on each irradiation day. A catheter was retrogradely placed from the superficial temporal artery to the maxillary artery to selectively deliver cisplatin to the tumor.

Resection of the primary tumor was performed with a safety margin of at least 10 mm, based on the assessment of tumor extent using physical examination and imaging findings. Reconstructive surgery using a vascularized free flap was performed for extensive skin, soft palate, or orbital floor. A wait‐and‐watch strategy was applied for the management of cN0. Patients with cN0 who received primary tumor excision and reconstruction with a vascularized free flap underwent END (levels I–III or I–V). Patients with clinically positive cervical lymph nodes underwent supraomohyoid neck dissection (levels I, II, and III) and radical or modified radical neck dissection at levels I–V.

Patients with a positive surgical margin, ≥ 4 pathological metastatic lymph nodes, or extranodal extension (ENE) with adhesion to the surrounding structures underwent postoperative RT with a fraction dose of 2 Gy to a total dose of either 50 or 60 Gy. When tolerated, it was combined with either concurrent oral administration of S‐1 on each irradiation day or tri‐weekly cisplatin (100 mg/m^2^). S‐1 is derived from 5‐FU and consists of tegafur, gimeracil, and oteracil potassium. The feasibility and efficacy of radiotherapy concurrent with S‐1 were demonstrated in oral cancer and the other cancers [22, 23, 24].

After radical treatment, patients were followed up every 4 weeks for 1 year, every 2 months for the next year, and every 3 months for another year, with gradually increasing follow‐up intervals thereafter. Surveillance included CT or FDG‐PET/CT performed 6 months post‐treatment and annually thereafter. The cervical lymph nodes were closely monitored through physical examination and US. The median follow‐up period was 78 months (interquartile range: 52–102 months).

### 2.3 Statistical Analysis

Pearson's chi‐square test or Fisher's exact test was used to assess the association between categorical variables and pathological CLNM. Multivariate logistic regression analysis was conducted to investigate the correlation between clinical features and pathological CLNM. For this analysis, categorical variables with a *p*‐value of less than 0.05 in the univariate analysis were selected as explanatory variables. Survival analysis was conducted using the Kaplan–Meier method, and between‐group comparisons were conducted using the log‐rank test. Five‐year disease‐specific survival (5‐year DSS) was selected as the outcome to focus on cancer‐specific prognostic factors affecting survival. The 5‐year DSS was defined as the period from the date of surgery to the date of death due to uncontrolled MGSCC. Multivariate analysis of factors associated with the 5‐year DSS was conducted using the Cox proportional hazards model. Variables with a *p*‐value of less than 0.01 in the log‐rank test were selected for inclusion in the Cox proportional hazards model analysis. A *p*‐value of < 0.05 indicated statistical significance. Interaction terms and multicollinearity were not assessed. JMP 14 and JMP 17 (SAS Institute Inc., Cary, NC, USA) were used for all statistical analyses.

## 3 Results

### 3.1 Characteristics of Patients, Treatments, and Outcomes

Table 1 presents the clinical and pathological characteristics of the 81 patients with MGSCC. The treatment for these patients includes surgery alone in 50 patients, preoperative RT (40–50 Gy) followed by surgery in 16 patients, and surgery followed by postoperative RT (50–60 Gy) in 15 patients. The surgical procedures for primary tumors included partial, subtotal, and total maxillectomy in 65, 14, and 2 patients, respectively. Neck dissection was performed in 28 patients, including END in two patients and therapeutic neck dissection in 26 patients. Pathological CLNM was observed in 24 patients.

**TABLE 1:** Clinicopathological characteristics of patients with maxillary gingival squamous cell carcinoma.

Clinicopathological factors	*n*
Age (median age: 75 years)	
< 75 years	40
≥ 75 years	41
Sex	
Male	40
Female	41
cT	
T1	22
T2	28
T3	4
T4	27
cN	
N0	65
N1	7
N2	9
Histologic grade	
Well	47
Moderate	29
Poor	5
Pathological CLNM	
Negative	57
Positive	24
Surgical margin status	
Negative	71
Positive	10
Preoperative treatment	
No	65
Yes	16
Postoperative treatment	
No	66
Yes	15
Local recurrence	
Negative	74
Positive	7
Prognosis	
No evidence of disease	69
Dead of diseases	9
Local failure	4
Regional failure	3
Distant failure	2
Dead of other causes	3

Abbreviation: CLNM: cervical lymph node metastases.

Seven patients experienced primary tumor recurrence during the follow‐up period. Among them, three patients who underwent salvage treatment achieved local control and survived. Among the four patients who died from uncontrolled primary recurrence, three had no CLNM. Regional recurrence was observed in three patients, all of whom eventually succumbed to the disease. Further, two patients died of distant metastatic disease despite achieving locoregional control. All five patients who died from regional recurrence or distant metastasis had CLNM. Three patients died from other causes.

### 3.2 Mode of CLNM

Among the 24 patients with CLNM, 11 had occult CLNM on the ipsilateral side. One patient exhibited ipsilateral CLNM in the END at the initial treatment, whereas 10 patients developed delayed ipsilateral CLNM. The frequencies of ipsilateral occult CLNM by cT stage were 13.6% (3/22 cases) for cT1, 14.3% (4/28 cases) for cT2, 0% (0/4 cases) for cT3, and 14.8% (4/27 cases) for cT4. Seven patients experienced CLNM on both sides. Among them, five demonstrated bilateral CLNM at the initial treatment, whereas two initially presented with ipsilateral CLNM and later developed contralateral delayed CLNM. No patient showed contralateral CLNM without ipsilateral involvement.

Table 2 summarizes the regions of the CLNM. Ipsilateral CLNM was predominantly observed in levels IB and II. In patients with ipsilateral CLNM, metastases were predominantly observed in levels IB and II, and there were no cases with metastasis to level III alone. Contralateral CLNM was mostly detected in level II. No patient had CLNM in levels IA, IV, and V on either side. The median number of ipsilateral CLNM was 2 (range: 1–7) and those of contralateral CLNM were also 2 (range: 2–6).

**TABLE 2:** Mode of cervical lymph node metastasis.

Level or region of the CLNM	Ipsilateral (*n* = 24)	Contralateral (*n* = 6^a^)
Level IA	0	0
Level IB	16 (67%)	1 (17%)
Level II	20 (83%)	5 (83%)
Level III	4 (17%)	2 (33%)
Level IV or V	0	0
Parotid node	0	1 (17%)

Abbreviation: CLNM: cervical lymph node metastases.

^a^ Among the seven patients with contralateral CLNM, one who did not undergo neck dissection because of the presence of distant metastasis was excluded from this analysis.

### 3.3 Prognostic Factors

The 5‐year DSS rate was 89.4% among all 81 patients. Table 3 presents the univariate analyses of the factors associated with 5‐year DSS. The results indicate that sex, preoperative treatment, and postoperative treatment were not statistically associated with DSS. In contrast, age, cT stage, histologic grade, CLNM, local recurrence, and surgical margin status were significantly associated with 5‐year DSS (*p* < 0.05). Among these factors, those with a *p*‐value < 0.01 were included in the multivariate analysis. The Cox proportional hazards model analysis identified histologic grade (hazard ratio [HR]: 8.42; 95% confidence interval [CI]: 1.37–51.93; *p* = 0.02), CLNM (HR: 7.09; 95% CI: 1.40–35.88; *p* = 0.02), and local recurrence (HR: 12.87; 95% CI: 2.58–64.27; *p* < 0.01) as independent prognostic factors for 5‐year DSS (Table 3). The results indicate that CLNM demonstrated a significant negative impact on the survival of patients with MGSCC.

**TABLE 3:** Statistical analyses of clinicopathological characteristics related to the 5‐year DSS.

Clinicopathological features	5‐Year DSS (%)		Log‐rank test	Cox proportional hazards model analysis		
			*p*	Hazard ratio	*p*	95% Confidence interval
Age						
≥ 75 vs. < 75 years	80.5	97.4	0.02			
Sex						
Female vs. male	87.5	91.8	0.56			
cT						
T3 + T4 vs. T1 + T2	78.2	95.8	0.03			
Histologic grade						
Poor vs. well + moderate	60.0	91.4	< 0.01	8.42	0.02	1.37–51.93
Pathological CLNM						
Positive vs. negative	72.8	96.4	< 0.01	7.09	0.02	1.40–35.88
Local recurrence						
Positive vs. negative	57.1	92.5	< 0.01	12.87	< 0.01	2.58–64.27
Surgical margin status						
Positive vs. negative	64.0	92.5	0.02			
Preoperative treatment						
Yes vs. no	80.2	91.8	0.18			
Postoperative treatment						
Yes vs. no	85.1	90.4	0.51			

Abbreviations: CLNM: cervical lymph node metastases; DSS: disease‐specific survival.

### 3.4 Predictive Factors for Pathological CLNM

We investigated the association between clinicopathological factors and CLNM. MGSCC can extend medially to the palate, laterally to the gingivobuccal sulcus, posteriorly to the maxillary tuberosity, and superiorly to the maxillary sinus. Therefore, we assessed the association between clinical tumoral spread to these anatomical sites and CLNM. The tumoral spread was diagnosed based on physical and imaging examinations or pathological evaluation. Univariate analysis revealed that age, sex, cT stage, tumoral spread to the median line, histologic grade, bone invasion, maxillary sinus invasion, and perineural invasion were not statistically associated with CLNM (Table 4). In contrast, a significant correlation was observed in tumoral spread to the gingivobuccal sulcus, tumoral spread to the maxillary tuberosity, and lymphovascular invasion with CLNM (*p* < 0.01, *p* = 0.02, and *p* = 0.04, respectively). The incidence of CLNM was 44.7% in 38 patients with primary tumoral spread to the gingivobuccal sulcus and 44.1% in 34 patients with primary tumoral spread to the maxillary tuberosity. Furthermore, multivariate analysis identified tumoral spread to the gingivobuccal sulcus (odds ratio [OR]: 3.41; 95% CI: 1.18–10.54; *p* = 0.02) and the maxillary tuberosity (OR: 2.83; 95% CI: 1.00–8.32; *p* < 0.05) as independent risk factors for CLNM (Table 5).

**TABLE 4:** Univariate analysis of the correlation between clinicopathological features and cervical lymph node metastasis.

Clinicopathological features	CLNM		*p*
	Positive (*n* = 24)	Negative (*n* = 57)	
Age			0.16
< 75 years	9	31	
≥ 75 years	15	26	
Sex			0.68
Male	11	29	
Female	13	28	
cT			0.36
T1 + T2	13	37	
T3 + T4	11	20	
Median line			0.17
Not	16	46	
Involved	8	11	
Gingivobuccal sulcus			< 0.01
Not	7	36	
Involved	17	21	
Maxillary tuberosity			0.02
Not	9	38	
Involved	15	19	
Histologic grade			0.15^a^
Well + moderate	55	21	
Poor	2	3	
Bone invasion			0.27
Negative or cortex	9	29	
Medulla	15	28	
Maxillary sinus invasion			0.64
Negative	16	41	
Positive	8	16	
Perineural invasion			0.63^a^
Negative	22	54	
Positive	2	3	
Lymphovascular invasion			0.04
Negative	16	49	
Positive	8	8	

Abbreviation: CLNM: cervical lymph node metastases.

^a^ Fisher's exact test.

**TABLE 5:** Multivariate analysis of the correlation between clinicopathological features and cervical lymph node metastasis.

Clinicopathological features	OR	*p*	95% CI
Gingivobuccal sulcus (involved vs. not)	3.41	0.02	1.18–10.54
Maxillary tuberosity (involved vs. not)	2.83	< 0.05	1.00–8.32
Lymphovascular invasion (positive vs. negative)	2.09	0.16	0.60–7.27

Abbreviations: CI: confidence interval; CLNM: cervical lymph node metastases.

## 4 Discussion

MGSCC is relatively rare, and its oncological behavior is not well understood. Further studies are needed to clarify the detailed characteristics and prognostic factors of patients with MGSCC. Previous studies have revealed that the prognostic factors for MGSCC include T stage [2, 12, 25], N status [16, 25], histologic grade [2], positive margin [25], and recurrence [25]. In the present study, the multivariate analysis revealed that CLNM had a strong negative effect on the prognosis of patients with MGSCC, comparable to poor histologic grade and primary tumor recurrence. Meanwhile, T stage and margin status were not considered independent prognostic factors for the 5‐year DSS. In the present study, there were nine cases of death due to MGSCC. Among the four patients who died from uncontrolled primary recurrence, three had no CLNM, while one had CLNM. In contrast, all five patients who died from regional recurrence or distant metastasis had CLNM. These results suggest that CLNM in MGSCC may be associated with further disease progression, namely regional recurrence and distant metastasis.

For many years, maxillary gingival carcinoma was considered to have a low potential for CLNM [9]. However, recent studies have reported that the incidence of CLNM in MGSCC is comparable to that in other oral SCCs [9]. In the present study, the incidence of CLNM was 29.6%, which is consistent with previous reports on maxillary SCC (ranging from 22.6% to 46.2%) and comparable to the incidence observed in our data for SCCs of the tongue (21.1% [26]), mandibular gingiva (25.4% [27]), and buccal mucosa (40% [28]), supporting this finding. Elective neck treatment is recommended for patients with head and neck SCC if the probability of occult CLNM exceeds 20% [29]. In the present study, occult CLNM was observed in 13.6% of patients, which is insufficient to justify recommending END for all patients with MGSCC. A previous study also reported that END is particularly recommended for patients with cT3/4 tumors due to the higher frequency of occult metastasis compared to those with early T‐stage tumors [9]. However, in the present study, no difference was observed in the frequency of occult metastasis between early and advanced cT‐stage tumors.

Several studies have investigated the risk factors for CLNM in MGSCC; however, only a few have conducted multivariate statistical analyses [11, 12, 13, 14, 15, 16, 17, 18, 19] (Table 6). To the best of our knowledge, the present study includes the largest number of MGSCC cases among these reports. In this study, multivariate analysis identified spread to the gingivobuccal sulcus and maxillary tuberosity as independent risk factors for CLNM. The external or buccal gingival lymphatic networks form several collecting vessels in the gingivobuccal sulcus [20]. These vessels pierce the buccinator muscle to follow the facial artery and vein, and then drain into the submandibular lymph nodes [20]. Tumors originating from the maxillary gingiva that extend laterally to involve the gingivobuccal sulcus may infiltrate this abundant lymphatic network, thereby increasing the risk of CLNM. Similar to the findings of the present study, the multivariate analysis conducted by Zhang et al. identified gingivobuccal sulcus involvement and poor histological differentiation as risk factors for CLNM, supporting this hypothesis [12]. The maxillary lingual gingival collecting vessels run posteriorly, blending with or joining the lymphatics of the hard and soft palate [20]. These lymphatics consist of three main pathways: anterior, middle, and posterior pathways [20]. Among these, the middle one is the most consistent pathway. This pathway runs posteriorly to reach the retromolar region, and it terminates in the upper internal jugular chain after traversing the medial aspect of the posterior belly of the digastric muscle [20]. The maxillary tuberosity is the most posterior portion of the maxillary alveolar ridge and is adjacent to the retromolar region and soft palate. Therefore, cancer cells extending to the maxillary tuberosity may infiltrate the middle pathway, thereby increasing the risk of CLNM.

**TABLE 6:** Summary of recent studies that statistically analyzed the risk factors of cervical lymph node metastasis in patients with maxillary squamous cell carcinoma.

	Year of published	Survey period	Statistical analysis	Number of MGSCC patients (all patients including hard palate)	Significant predictive factors of pathological CLNM	
					Univariate analysis	Multivariate analysis
Ogura et al.	2003	1994–2000	Multi	21 (21)		Maxillary bone invasion
Beltramini et al.	2012	2000–2010	Uni	37 (65)	None	
Yang et al.	2014	2002–2011	Uni	34 (71)	T stage, vascular invasion	
Sagheb et al.	2014	1987–2011	Uni	Not described (135)	T stage, histologic grade Tumor location (pre‐ vs. post‐canine)	
Berger et al.	2015	1975–2009	Uni Multi	32 (171)	Age, T stage	
Zhang et al.	2015	2000–2010	Uni Multi	70 (100)	T stage, histologic grade Involving the gingival buccal sulcus	Histologic grade (I vs. III) Involving the gingival buccal sulcus
Troeltzsch et al.	2016	2006–2013	Uni	39 (92)	Age, gender, histologic grade	
Moratin et al.	2018	1999–2016	Uni	Not described (68)	T stage, tumor differentiation, lymphatic infiltration	
Present study		2008–2018	Uni Multi	81 (81)	Spread to the gingivobuccal sulcus Spread to the maxillary tuberosity Lymphovascular invasion	Spread to the gingivobuccal sulcus Spread to the maxillary tuberosity

Abbreviations: CLNM: cervical lymph node metastasis; MGSCC: maxillary gingival squamous cell carcinoma; Multi: multivariate analysis; Uni: univariate analysis.

Some studies have identified the T stage as a risk factor for CLNM [11, 12, 13, 15, 16]; however, it was not found to be a risk factor in the present study. Further, among the T1 and T2 cases, the incidence of CLNM was higher in tumors that extend to the gingivobuccal sulcus or maxillary tuberosity (58.3% or 36.8%, respectively) than in those without such extension (15.8% or 19.4%, respectively) (data not shown). Altogether, these results suggest that even small tumors may have a higher potential for CLNM if they extend into the gingivobuccal sulcus or maxillary tuberosity.

Previous studies on CLNM sites in MGSCC have reported that metastases most commonly occur in levels I and II [4, 12, 30]. Similarly, in the present study, all patients with ipsilateral CLNM had metastases involving level IB, level II, or both; no cases showed metastasis to level III alone. Ipsilateral CLNM was observed in level IB in 67% of cases and in level II in 83%. Furthermore, among the 24 pN+ cases, 76.5% of tumors that invade the sulcus involved level IB, whereas 93.3% of tumors invading the tuberosity included level II (data not shown). These findings may reflect the aforementioned lymphatic drainage from the maxillary gingiva. Contralateral CLNM occurred in seven patients (29.2%) in the present study, and no cases showed contralateral metastasis without ipsilateral CLNM. The lymphatic drainage from the palatal gingiva and palate merges along its pathway, with crossing lymphatic vessels near the midline, draining into the contralateral upper jugular lymph nodes [20]. These crossing vessels are especially significant in areas with abundant minor salivary glands [20]. The palatal glands are located on the palate posterior to the line connecting both first molars, where glandular tissue is particularly abundant in the posterior hard and soft palate. Therefore, tumors that extend to the midline, or even those that do not reach the midline but extend posterior to the molars into areas containing palatal glands, are thought to have the potential to spread along the contralateral lymphatic flow. Of the seven patients with contralateral metastasis in this study, six had tumors that spread to the midline or into the maxillary tuberosity (data not shown). Additionally, 83.3% of patients with contralateral CLNM had metastases to contralateral level II. Taken together, although statistical analysis was not performed, these findings suggest that attention should be paid to contralateral level II metastasis in MGSCC patients whose primary tumor extends to the midline or into areas containing palatal glands, and who also have ipsilateral CLNM.

A limitation of this study is the relatively small sample size, which may reduce the statistical power of the analysis. To address this limitation and validate the findings in a larger patient cohort, thereby enhancing generalizability, a multi‐institutional collaborative study with a larger sample size is warranted.

## 5 Conclusion

CLNM is associated with poor survival outcomes in patients with MGSCC. Primary tumoral spread to the gingivobuccal sulcus or maxillary tuberosity is considered a risk factor for CLNM. Regardless of the T stage, tumors with such extension should raise concern for CLNM, particularly in levels IB and II. These findings provide valuable insights for optimizing neck management strategies in MGSCC patients.

## Ethics Statement

This study complied with the Declaration of Helsinki and obtained approval from the Institutional Review Board of Tokyo Medical and Dental University (D2015‐600).

## Consent

Informed consent was obtained in the form of opt‐out on the in‐hospital bulletin.

## Conflicts of Interest

The authors declare no conflicts of interest.

## Data Availability

The data that support the findings of this study are available from the corresponding author upon reasonable request.
